# Impact of the COVID-19 pandemic on medical office assistants (MOAs) working in primary care: a qualitative study

**DOI:** 10.3399/BJGPO.2024.0151

**Published:** 2025-05-07

**Authors:** Jennifer K Johnson, Bridget L Ryan, Amanda L Terry, Judith Belle Brown

**Affiliations:** 1 Department of Family Medicine, Schulich School of Medicine and Dentistry, Western University, Ontario, Canada; 2 Department of Family Medicine, Department of Epidemiology and Biostatistics, Schulich School of Medicine and Dentistry, Western University, Ontario, Canada; 3 Department of Family Medicine, Department of Epidemiology and Biostatistics, Schulich Interfaculty Program in Public Health, Schulich School of Medicine and Dentistry, Western University, Ontario, Canada; 4 Department of Family Medicine, Schulich School of Medicine and Dentistry, Western University, Ontario, Canada

**Keywords:** qualitative research, family medicine, professions allied to medicine (PAMs), primary healthcare, general practice, medical receptionists, medical office assistants

## Abstract

**Background:**

Medical office assistants (MOAs), also known as receptionists and clerks, are frontline workers and the most accessible member of the primary care team. Historically, their contributions to primary care have been unrecognised and undervalued. The COVID-19 pandemic put pressure on existing roles and systems in primary care: how MOAs adapted is unknown.

**Aim:**

To explore the experiences of MOAs working in primary care during the COVID-19 pandemic from the perspectives of MOAs and family physicians (FPs) who worked with MOAs during this period.

**Design & setting:**

A qualitative study, using constructivist grounded theory (CGT), was conducted in Ontario, Canada.

**Method:**

Seventeen participants were recruited through professional contacts of the research team. Individual semi-structured interviews were undertaken with MOAs and FPs across the province.

**Results:**

MOAs’ many responsibilities in primary care intensified during the pandemic. MOAs leveraged their healthcare system knowledge and therapeutic relationships with patients to reduce patient distress. Unfortunately, MOAs experienced more frustration, and in some cases, abuse from patients. MOAs’ ability to adapt to new systems and respond to high patient needs seemed to be positively influenced by their relationships with patients and FPs. FPs showed support for MOA welfare and recognised their critical role on primary care teams.

**Conclusion:**

MOAs made considerable contributions to primary care during the COVID-19 pandemic. This study suggests MOAs have greater capacity than previously recognised, which has important implications for planning in an era of under-resourced health care.

## How this fits in

To our knowledge, this is one of the first qualitative studies to explore the experiences of medical office assistants (MOAs) during the COVID-19 pandemic. Our study provided a window into a healthcare system under acute stress and confirmed all the known multiple necessary elements from the literature that promote and support high-functioning primary care MOAs. However, what we learnt from MOAs who worked through this crisis is that they have unrecognised capacity and innovation, which has resonance with and is applicable to MOAs working in the current climate of under-resourced primary care. This study highlighted their essential role of providing patient-centred care and that MOAs cannot easily be supplanted by novel initiatives to improve health system efficiency in the future.

## Introduction

MOAs are frontline workers and the first contact for patients seeking primary care. The term MOA refers to medical office receptionists, clerks, and medical administrative assistants who have direct contact with patients.

Canada’s publicly funded healthcare system is accessed through primary care, which provides comprehensive health care from birth to death.^1^ Canadian MOAs are hired for primarily administrative roles in medical offices and often learn on the job. The proportion of MOAs with formal training is difficult to determine as they are not registered with a Canadian licensing body.^2^ Similarly, there are no specific training requirements for MOAs in Australia,^3^ Ireland,^4^ and the US.^5^ However, the NHS in the UK has mandated online education in triage and patient safety for general practice administrative staff.^6^

MOAs significantly influence patient access to, and utilisation of services.^7,8^ As gatekeepers to primary care, they are responsible for triage decisions that affect patient care, yet they have little medical training or control over practice processes and workflow.^7,8^ Historically, the contributions of MOAs to primary care have been unrecognised and undervalued.^8,9^ Most MOAs are women and are poorly paid with an average wage of $20.00 CND (approximately 11 GBP) per hour in Ontario.^10^

COVID-19 drastically changed how primary care was delivered. Operational pressures on primary care included having to secure personal protective equipment (PPE), triage patients to in-person versus virtual consultations and navigate a changing healthcare system.^11^ It is probable, but unknown, if what was experienced by family physicians (FPs) and nurses during COVID-19^12^ was also experienced by MOAs. The aim of this study was to understand the experiences of MOAs during the COVID-19 pandemic. Insights were gained from both MOAs and FPs working in primary care in Ontario.

Given the paucity of research conducted on this occupational group, and their recognised value to primary care,^13^ it is important to understand how MOAs fared with the unprecedented challenge of a global pandemic. Evidence from this study may inform planning for optimising team-based care now, and for future catastrophic events.

## Method

### Study design

This qualitative study was conducted using constructivist grounded theory (CGT).^14^ CGT is a process of data collection and analysis that explains how and why participants construct meanings and actions in the context of social processes, such as the dynamic changes to clinic staff experiences during the COVID-19 pandemic.

### Recruitment and sampling

Primary care clinics across Ontario were contacted by publicly available means to recruit interested MOA and FP participants. Posters were mailed to clinics and posted on Canadian MOA and FP Facebook social media groups along with a promotional video featuring the principal investigator (PI; JJ) explaining the study. Participants were also recruited from professional contacts of the research team.

Participants were informed that the PI (JJ) was an FP. Six of the FP participants and two of the MOA participants were known to the PI. None of these participants had worked with the PI.

The final sample size of 17 was based on saturation of themes. All MOAs and FPs who were interested participated in the study.

### Data collection

Interviews were conducted between October 2022 and June 2023 by JJ using two separate semi-structured interview guides. The MOA interview guide was developed with input from study collaborators who are MOA educators from Georgian College, Ontario, Canada. Each interview guide broadly covered the topics of MOA responsibilities, challenges, job satisfaction, relationships with patients and clinic staff during the pandemic, changes to MOAs’ contributions to primary care, and how MOAs could be best supported in the future (Supplementary Box 1).

Interviews were audio-recorded in person or through the online virtual meeting platform, Zoom. Demographic data collected included age, sex, race category, and years working as an MOA or FP.

Participants were asked how the efforts of MOAs were recognised and appreciated by FPs and how to prepare future MOAs for this occupation. The interviews were 30–60 minutes. Each participant was given a $30 CND (approximately 17 GBP) grocery store gift certificate.

### Data management

Interviews were transcribed verbatim by a professional transcription service and input into the qualitative data management software program, NVivo (versions 12 and 14).

### Data analysis

During and following the interviews, JJ made reflective field notes. The transcribed interviews were reviewed for accuracy and then analysed first individually, and then together by the research team. The transcripts were analysed line by line to develop initial codes. At regular meetings, the research team (JJ, BR, AT, JBB) implemented focused coding by synthesising and conceptualising the initial codes into those that best reflected the data. Varying points of view during analysis were discussed and an agreed on interpretation was determined after extensive discussion of all team members’ perspectives. The modified coding was applied in subsequent interviews. As more data were collected, the team looked for connections between codes, integrating them into categories that illuminated the research question and reflected the emerging theory. In the final analysis stage, the team considered how the categories related to each other, grouping them into higher order themes. Constant comparison and reflection were used throughout the analysis consistent with CGT. This iterative process of simultaneous data collection and analysis was documented with memos and continued until saturation of the themes was achieved.^15,16^ In keeping with CGT, and to promote trustworthiness, team members practised reflexivity. Possible biases and previous experiences that they brought to their interpretation of the data based on their backgrounds (family medicine, epidemiology, and social work) were constantly examined from data collection to manuscript preparation.^14^ The PI has some understanding of the challenges facing MOAs from her clinical work as a FP, which sensitised her to the issues raised by participants.

## Results

### Study population

Seventeen participants were recruited to the study: eight MOAs and nine FPs. The MOAs identified as female, belonging to Indigenous and White race categories, and ranged in age from 32–57 years. Years working as an MOA ranged from 5–26.

FP participants were comprised of six women and three men from Asian, South Asian, Middle Eastern, and White race categories. The FPs ranged in age from 40–64 years, with years in practice ranging from 6–32.

### Findings

#### Overview

The team analysed the MOA transcripts and the FP transcripts separately, and as part of the constant comparison process, examined similarities and differences between the two groups. Analysis of the interviews with MOA and FP participants revealed multiple perspectives in accordance with CGT: MOAs have critical skills and relationships that they use in supporting primary care. The COVID-19 pandemic made this abundantly evident. During this tumultuous time, the participants explained that patients’ healthcare needs increased, resources were often limited and that MOAs needed to stay abreast of constantly changing COVID-19 information and public health restrictions. Participants described staff health risks and shortages, and pivoting to virtual care. Overall, the demands on MOAs increased, necessitating expansion and realisation of their roles facilitating primary care.

The following five themes were identified during analysis: navigating uncharted territory; stepping up; fortitude and compassion towards patients; nurturing patient–MOA relationships; and nurturing FP–MOA relationships.

#### Navigating uncharted territory

MOA and FP participants explained that even during COVID-19 lockdowns, *’we were really accessible to our patients’* (MOA#7). However, resources such as PPE, in-person appointments, and consultations with specialists were often insufficient during the pandemic. Securing health care for patients became more challenging:

*’ …* [cancelling surgeries] *increased the burden on primary care … there* [was] *nowhere for patients to turn.* [MOAs were] *getting longer phone calls, more frustration, more multi-issue visits …’* (FP#3).

According to MOA and FP participants, more patients presented with advanced disease during and after the COVID-19 pandemic lockdowns. This caused more stress for MOAs:

*’I’ve been here for how many years? You have people that are coming into us* [for their first visit] *that are* [already] *palliative … that is a hard pill to swallow’* (MOA#3).

MOAs had to use their skills navigating the healthcare system quickly for these patients:

*’I’m that liaison between the doctor and the patient … I know how things work, and I’ve been doing it for so long I can … make things move faster if I need to.’* (MOA#1).

Study participants felt that the MOA workload increased over this time:

*’Everyone is pretending that the pandemic is over …, but I don’t think it has … the work is quite heavy and demanding, more so than before the pandemic’* (FP#7).

#### Stepping up

MOAs are the first contact for patients seeking health care in the community. They perform a vast number of tasks and manage much of the administrative load in a primary care office:

*’… you’re answering the phones, you’re checking in people, you’re keeping track of all the appointments, keeping track of even the patient’s specialty appointments, notifying the patients about those, nagging the specialists to try and get appointments for them. We’re scanning and getting all the reports from all the specialists and doctors and tests that people have done, putting those in the chart, notifying the doctors ...’* (MOA#5)

Many FP participants were impressed that MOAs *’took a really significant leadership role’,* to reduce the spread of COVID-19: *’*[MOAs] *completely took ownership of* [the waiting room] *space and laid out what they had planned’* (FP#3). MOAs found it stressful *’knowing whether or not patients were urgent enough to bring into the clinic … making sure everybody’s got their PPE on*’ and ensuring that *’the room has been sanitised before and after’* (MOA#7).

MOAs also had to respond quickly to public health and FP dictated changes:

*’I don’t blame public health … everything was evolving all the time. But you’d be explaining the rules on the phone one day and by the afternoon we were getting a notice saying, "okay, now it’s like this".’* (MOA#5).

Many MOAs were invited to COVID information sessions for the first time:

*’Our department gave a weekly COVID update .. .and lots of the MOAs, people who wouldn’t have gone to rounds, would sign in’* (FP#7)

MOA participants expressed enthusiasm for access to comparable resources in the future:

*’I would love … specific training for epidemics. How can we prepare for something of this magnitude?’* (MOA#2).

Several participants remarked that MOAs also helped organise and manage the COVID-19 vaccination effort during the pandemic:

*’It took a lot of work to make those things* [vaccines] *happen smoothly. And they* [MOAs] *really stood up to take a good part … of that’* (FP#4).

#### Fortitude and compassion towards patients

During the COVID-19 pandemic, the work of MOAs intensified. Not only did MOAs communicate important information about masking, testing, self-isolation, and COVID vaccines, but also MOAs spent more time on the phone defusing patient frustrations:

*’… sometimes it’s just a matter of being patient with* [patients]*, hearing them out, letting them have their little moment and then reiterating the important things, they calm down’* (MOA#5).

Unfortunately, both MOAs and FPs described instances of abuse by patients:

*’Over the pandemic, someone … reached through the window and grabbed her* [MOA co-worker] *on the shoulder, because they were mad … People say things like, "It’s your fault if I die" … we’ve had people verbally swear at us’* (MOA#1).

FPs echoed this observation:

*’… the patients are becoming impatient … and sometimes a little abusive to our MOAs’* (FP#8).

MOA participants found interactions with upset patients common and very challenging. They explained that much of their work is *’about managing people and their expectations’* and not *’taking it personally’* when *’*[patients] *are having a bad day’* (MOA#8). They also expressed a need for *’de-escalation training’* to be prepared for *’all the personalities … we’re going to encounter’* (MOA#4), and to know *’how you have to react* [to conflict]*, so that you’re going to leave on a good note’* (MOA#5).

Despite these negative experiences, the MOA participants expressed that *’patients are probably my number one’* (MOA#2) and that *’just helping people’* was *’really rewarding’* (MOA#8). MOA participants also observed that patients who were isolated in the pandemic: *’reached out more … some of them … calling for nothing … "Am I due for something?" when they weren’t … I think it was loneliness, just to hear a voice’* (MOA #3).

MOAs helped relieve some of the suffering during the pandemic: *’… the empathy, the kindness ... I hear the* [MOAs] *… telling the patients with COVID to look after themselves and if there’s any concerns to phone us. They really stepped up’* (FP#1).

#### Nurturing patient–MOA relationships

Despite not being regulated or licensed healthcare professionals, MOAs have a clinical role on primary care teams. Their actions and attention are often therapeutic:

*’… Patients still ask after her and … miss her. I think it just speaks to the* [fact] *that these are not administrative roles. They are not clerical jobs.* [MOAs] *have a very therapeutic presence for people’* (FP#7)

MOA participants explained that this important relationship develops over time by knowing patients and consistently responding to their needs:

*’ … I’ve had a relationship with* [most patients] *for a long time, a working relationship as their secretary … I can … bring a sense of calm, or say, "I’m going to help you get what you need," which kind of makes them feel better.’* (MOA#1)

FP participants acknowledged that *’MOAs have formed relationships* [with patients] *too’* and that MOAs *’know our patients as well as anybody else … in our office’* (FP#4)*,* leading one to propose that *’continuity* [with] *MOAs is probably equally important as continuity with your provider’* (FP#2).

One of the FP participants articulated their view of the MOA’s role:

*’Some of the good that is done within family medicine is that piece of knowing and being known and sort of being met with unconditional positive regard … The medium through which they* [MOAs] *do their work is the front desk. The medium through which I do the work is by being the doctor in the room, but at the end of the day, we are both doing the same work.’* (FP#7)

#### Nurturing FP–MOA relationships

The stress of the pandemic led both MOA and FP participants to appreciate the importance of their working relationship. For example, MOAs know that FPs have limited time and attention to give their patients:

*’Patients know that they can reach out to us because sometimes, or most of the time, we can help them out’,* instead of *"interrupting the physician".’* (MOA#3).

FP participants appreciated that their MOAs took responsibility for reducing everyone’s exposure to the virus: *’I still hear them* [the MOAs] *now, "Do you have a cough? ... Have you done a COVID test?" They’re an extra layer of protection for us’* (FP#1). In kind, most FP participants extended protection to their MOAs: *’I’m the one seeing patients ... Why don’t we keep exposure limited … behind the plexiglass, so you … feel safe?’* (FP#6).

However, not all MOA participants felt adequately protected by their FP colleagues during the pandemic: *’The doctors had N95 masks, but we never ever got N95 masks’* (MOA#5). Additionally, they were not warned when a patient who had *’tested positive for COVID’,* was sent to the front desk *’to spend 5 minutes doing stuff with* [us]*’* (MOA#5).

Some FPs showed support for MOAs by permitting them to take *’a mental health day’* if needed (MOA#3). Most MOAs felt that their ideas and concerns were heard: *’There was nothing that we could not say … We were all in this* [the pandemic] *for the first time … we were told speak freely’* (MOA#3). After attending COVID educational sessions, MOAs helped explain *’public health policy’* and *’vaccine eligibility’* to patients, which resulted in *’a flattening of hierarchy’* (FP#3) in the MOA–FP relationship.

An MOA participant remarked that being appreciated by the FP they worked with gave them *’the oomph to want to get up every day and go to work’: ‘I’m blessed that the doctor … tells us … how great we are … That is a big thing for me to be validated,* [that] *what I’m doing is helping everybody involved’* (MOA#1).

The majority of FPs acknowledged MOAs as indispensable and expressed deep appreciation for their skills and knowledge:

*’If we didn’t have them, we couldn’t open the offices, right? Even if you’re available, without your MOAs you couldn’t work’* (FP#9).

The relationship between FPs and MOAs emerged as essential to both parties and became stronger through their united efforts during the pandemic. MOAs shared that they would feel more supported in the future *’if MOAs were heard and their opinions were valued’* with *’more debriefing at the end of the day, and* [FPs] *saying, "What can I do to make your life easier?"’* (MOA#1).

## Discussion

### Summary

COVID-19 catalysed MOAs to meet the unique challenges associated with the pandemic including the following: navigating the sudden increase in healthcare system complexity and scarcity; nurturing their relationships with patients to reduce patient fear and isolation; and nurturing their relationships with FPs to optimise primary care delivery. MOAs understood that patients needed their help to navigate a complex, changing healthcare system and to be given important information and reassurance during this difficult time. MOAs also stepped up and managed new virtual and physical office spaces and often assisted in setting up vaccine clinics. Unfortunately, MOAs had to absorb more frustrations and, in some cases, abuse from patients. Despite the fortitude of MOAs, these experiences brought home the need for tools to prevent and manage conflict with patients.

Analysis of the participant interviews revealed that the essential aspects of the FP–MOA relationship — of being respected, valued, and recognised for their work — were necessary for MOAs to realise their full potential on primary care teams. Efforts made by FPs to include MOAs in learning about COVID-19 and in planning new clinic processes not only made MOAs feel valued but also may have mitigated negative responses to the increased demands of patients.

This study provided a window into a healthcare system under acute stress during the COVID-19 pandemic. What we learnt about the capacity and innovation of MOAs from this study has resonance and is applicable to MOAs working in the current climate of under-resourced health care.

### Strengths and limitations

This study’s main strength is examining the experiences of MOAs, an understudied group, at a time when primary care was under tremendous pressure from the COVID-19 pandemic. To our knowledge, this is among one of the first qualitative studies to explore the experiences of MOAs during the pandemic.

The limitations of this study are not being able to recruit any MOAs who left primary care during the pandemic, as their experiences of being an MOA during this time could have added important insights. MOAs are known to be a difficult-to-reach research population.^17^

It was noted that some MOA participants did not express many feelings in response to the stressors brought on by the pandemic. Perhaps this group did not reflect on their emotional reactions based on the interviewer’s questions, or they were not used to sharing personal responses to the difficulties they face, as their work often requires emotional neutrality.^18^A US study similarly found that clerks did not easily self-reflect on their work lives.^19^

### Comparison with existing literature

Previous research concluded that high-functioning MOAs add significant value to primary care.^13^ The COVID-19 pandemic brought this into sharper focus because the role of MOAs on primary care teams intensified during this difficult time.

#### 1. Navigating uncharted territory

As gatekeepers to primary care, MOAs have considerable influence on how patients experience health care, often engaging in clinical activities such as triage for which they have little or no training.^20^ During the pandemic, MOAs had to navigate limited healthcare services, which was especially difficult for patients who presented with advanced disease during and after lockdowns. MOAs routinely witness the gap between what patients need and what can be provided.^8^ Our study revealed that this mismatch grew during COVID-19. Despite these challenges, FPs reported witnessing MOAs consistently striving to help patients and team members during this time.

#### 2. Stepping up

MOAs make triage decisions that directly affect patient care and outcomes.^7^ This triage responsibility intensified during the pandemic: during the lockdowns, many MOAs had to decide not just when a patient should be seen but, often, which patients warranted a potentially risky in-person visit versus a virtual assessment. This is in keeping with the trend of MOAs being increasingly depended on for clinical roles even though they are not reimbursed or trained for these functions.^21^

The upheaval of the COVID-19 pandemic forced MOAs to establish and adapt to new processes for patients seeking primary care. MOA participants found it challenging to quickly implement these novel approaches, especially in the context of a strained healthcare system. MOAs often deal with the consequences of systems without the ‘*autonomy or status to overhaul them*’,^22^ yet they are ‘*key when considering the introduction of new systems for how patients access care*’.^23^

The MOAs, like other Canadian healthcare workers, experienced an increased workload, and having to do work they do not normally do during the pandemic.^24^

In our study, the majority of MOAs participated in organising new processes and systems: they ‘stepped up’ by helping plan vaccine clinics and took initiative to set up safe patient flow through waiting and examining rooms. Involving MOAs in these processes may have enhanced how primary care functioned in the pandemic.

#### 3. Having fortitude and compassion towards patients

Seeking more information from patients to make difficult triage decisions during COVID-19 may have had implications. Before the pandemic, research found that MOAs who ask for presenting complaints sometimes experience hostility from patients.^25^ In screening for COVID-19, MOAs had to gather extra information from patients that may have contributed to the reported increase in patients’ hostile behaviours. Furthermore, communication almost exclusively via the phone or through other virtual means may have contributed to MOAs misunderstanding of some patient concerns since digital technologies may risk losing the contextual understanding of individual patients by receptionists.^26^

A systematic review of aggression towards receptionists by Willer *et al,* ^22^ concluded that aggression was unfortunately a ‘*frequent and routine occurrence in general practice*’^22^ and that MOAs experienced emotional exhaustion maintaining composure in these situations. Our study findings are in keeping with this: all MOA participants described difficulty managing adversarial encounters with patients and highlighted that these confrontations had not only increased during the pandemic, but also were ongoing, seemingly fuelled by demand for primary care services outstripping supply.

MOA participants also noted an increase in patient calls for reassurance during the pandemic: this has previously been recognised as a responsibility that often falls on MOAs.^21^ MOAs in this study strove to meet this escalating need during the pandemic by using their listening skills and established relationships with patients.

#### 4. Nurturing patient-MOA relationships

With regard to nurturing patient–MOA relationships, during the pandemic, MOAs spent considerable time assisting patients. MOAs’ responses to patients’ complex needs both on the phone and in the waiting room^21^ can create a sense of personalised care,^27^ and lead to the optimal relational outcomes of trust, hope, and a sense of being known.^28^ All study participants understood the therapeutic value of MOAs’ long-term relationships with patients, a finding consistent with previous research.^13^

Also, in keeping with previous research,^21^ the MOA participants in this study felt that providing care to patients was the most satisfying of all their responsibilities.

#### 5. Nurturing FP-MOA relationships

Regarding nurturing FP-MOA relationships, MOA participants remarked that they were invited to team meetings and information sessions about COVID-19 for the first time. This practice of including MOAs in team meetings has previously been recognised as important not only because MOAs can provide valued feedback about how policies affect them,^19^ but also they have established knowledge and rapport with individual patients,^13^ which can enhance patient-centred care.^19^

MOA participants valued receiving recognition for their efforts by FP colleagues, inspiring them to work to the top of their abilities. Meeting and troubleshooting around novel challenges during the pandemic meant MOAs were able to share with FPs what was happening in their role. MOA satisfaction, not reducing work hours^29^ and retention in COVID-19^^12^^ have been found to be positively correlated with support and appreciation from FPs. Eisner and Britten suggested that FPs developing a greater understanding of receptionists’ work helps FPs realise MOAs’ commitment to patients, physicians, and practices.^20^

The authors of the present study believe that striving to provide primary care together during COVID-19 increased FPs’ awareness of the value of MOAs.

Our study is unique in being one of the first studies of MOAs in Canada, and by including FPs’ perspectives on MOAs working in primary care during the COVID-19 pandemic. We believe that our study not only confirmed the multiple necessary conditions that promote and support high-functioning MOAs, but also importantly highlighted what MOAs are capable of in a crisis. Increased inclusion and teamwork were essential during this time as were understanding and meeting greater patient needs. This study is proof of concept. All previous known elements from the literature coalesced in this study reflecting the entire role of MOAs as one lived experience. MOAs made significant contributions to providing primary care during a global pandemic, which suggests MOAs may have greater capacity than previously recognised. This has important implications for primary care planning in an era of great challenges to healthcare organisations.

### Implications for research and practice

The value of MOAs to primary care was illuminated in this study. Their relationships with FPs strongly influenced the ability of MOAs to function optimally in their multiple roles. This study reinforces the importance of including MOAs in team meetings and serves as a reminder to primary care providers that recognising MOA contributions and seeking their input on office processes can enhance MOA satisfaction and primary care delivery.

MOA relationships with patients enhance the delivery of patient-centred primary care as well. Our study highlighted their essential role: MOAs cannot easily be supplanted by novel initiatives to improve health system efficiency in the future.

MOA participants in this study identified the need for training to be able to de-escalate belligerent patients. This need is not currently addressed in Ontario college programme curriculums.^30^ Research suggests this kind of training has been effective ‘*in reception staff feeling safer and more confident dealing with hostile behaviours*’.^22^ It is strongly recommended that curricula like this be implemented and evaluated in Canada.

Currently, primary care in Canada is under tremendous strain, with approximately 22% of the population without a primary care provider.^31^ The heavy administrative workload is cited as one of the main reasons for FPs retiring early and medical school graduates not choosing this specialty.^32^ Future studies to investigate whether MOAs could help with these FP administrative responsibilities are worth considering. Other countries, such as the UK and Australia, are increasing MOA training and qualifications.^7,21,33^ However, determining the impact of these initiatives on the wellbeing and workload of FPs and MOAs would be essential.

Future survey studies to determine the wellbeing of MOAs would be helpful for health force planning.

## Data

The research data are not shared because they contain sensitive and potentially identifying information.

## References

[1] Government of Canada (2019). Canada’s health care system. https://www.canada.ca/en/health-canada/services/health-care-system/reports-publications/health-care-system/canada.html#a9.

[2] Government of Canada (2024). Medical office assistant in Canada. https://www.jobbank.gc.ca/marketreport/requirements/24389/ca.

[3] HCNet Training requirements for medical receptionists in Australia: understanding the landscape. https://generalpracticetraining.com.au/training-requirements-for-medical-receptionists-in-australia-understanding-the-landscape.

[4] Atkinson-Dalton E (2023). How to become a medical receptionist in Ireland. https://www.allcourses.ie/blog/how-to-become-a-medical-receptionist-in-ireland/.

[5] Teal (2024). Education requirements for medical receptionists. https://www.tealhq.com/education/medical-receptionist.

[6] NHS England (2023). Administrative triage using digital tools in general practice. https://www.e-lfh.org.uk/programmes/.

[7] Litchfield I, Gale N, Burrows M, Greenfield S (2017). The future role of receptionists in primary care. Br J Gen Pract.

[8] Neuwelt PM, Kearns RA, Browne AJ (2015). The place of receptionists in access to primary care: challenges in the space between community and consultation. Soc Sci Med.

[9] Hammond J, Gravenhorst K, Funnell E (2013). Slaying the dragon myth: an ethnographic study of receptionists in UK general practice. Br J Gen Pract.

[10] Statistics Canada (2020). Primary health care providers, 2019. https://www150.statcan.gc.ca/n1/pub/82-625-x/2020001/article/00004-eng.htm.

[11] Khalil-Khan A, Khan MA (2023). The impact of COVID-19 on primary care: a scoping review. Cureus.

[12] Wisbey K, Lane R, Neil J (2024). Leading primary care under the weight of COVID-19: how leadership was enacted in six Australian general practices during 2020. Aust J Prim Health.

[13] Solimeo SL, Stewart GL, Rosenthal GE (2016). The critical role of clerks in the patient-centered medical home. Ann Fam Med.

[14] Charmaz K (2014). Constructing grounded theory.

[15] Braun V, Clarke V (2019). Reflecting on reflexive thematic analysis. Qual Res Sport Exerc Health.

[16] LaDonna KA, Artino ARJ, Balmer DF (2021). Beyond the guise of saturation: rigor and qualitative interview data. J Grad Med Educ.

[17] Burrows M, Gale N, Greenfield S, Litchfield I (2020). A quantitative assessment of the parameters of the role of receptionists in modern primary care using the work design framework. BMC Fam Pract.

[18] Ward J, McMurray R (2011). The unspoken work of general practitioner receptionists: a re-examination of emotion management in primary care. Soc Sci Med.

[19] Solimeo SL, Ono SS, Stewart KR (2017). Gatekeepers as care providers: the care work of patient-centered medical home clerical staff. Med Anthropol Q.

[20] Eisner M, Britten N (1999). What do general practice receptionists think and feel about their work?. Br J Gen Pract.

[21] Neuwelt PM, Kearns RA, Cairns IR (2016). The care work of general practice receptionists. J Prim Health Care.

[22] Willer F, Chua D, Ball L (2023). Patient aggression towards receptionists in general practice: a systematic review. Fam Med Community Health.

[23] Brant HD, Atherton H, Bikker A (2018). Receptionists’ role in new approaches to consultations in primary care: a focused ethnographic study. Br J Gen Pract.

[24] Statistics Canada (2022). Experiences of health careworkers during the COVID-19 pandemic, September to November 2021.

[25] Iacobucci G (2016). Discussing symptoms with receptionists may put patients off seeing their GP. BMJ.

[26] Litchfield I, Burrows M, Gale N, Greenfield S (2022). Understanding the invisible workforce: lessons for general practice from a survey of receptionists. BMC Prim Care.

[27] Brown EJ, Kangovi S, Sha C (2015). Exploring the patient and staff experience with the process of primary care. Ann Fam Med.

[28] Scott JG, Cohen D, Dicicco-Bloom B (2008). Understanding healing relationships in primary care. Ann Fam Med.

[29] Sinsky CA, Brown RL, Stillman MJ, Linzer M (2021). COVID-related stress and work intentions in a sample of US health care workers. Mayo Clin Proc Innov Qual Outcomes.

[30] Ministry of Training Colleges and Universities (2015). Office administration—health services program standard.

[31] Duong D, Vogel L (2023). National survey highlights worsening primary care access. CMAJ.

[32] Pauls K (2023). Medical students interest in being family doctors on decline even as provinces boost training spots.

[33] Litchfield I, Gale N, Burrows M, Greenfield S (2023). “Y*ou’re only a receptionist, what do you want to know for?*”: street-level bureaucracy on the front line of primary care in the United Kingdom. Heliyon.

